# hATTR Pathology: Nerve Biopsy Results from Italian Referral Centers

**DOI:** 10.3390/brainsci10110780

**Published:** 2020-10-26

**Authors:** Marco Luigetti, Marina Romozzi, Giulia Bisogni, Davide Cardellini, Tiziana Cavallaro, Andrea Di Paolantonio, Gian Maria Fabrizi, Silvia Fenu, Luca Gentile, Marina Grandis, Gianluca Marucci, Sara Massucco, Anna Mazzeo, Davide Pareyson, Angela Romano, Massimo Russo, Angelo Schenone, Matteo Tagliapietra, Stefano Tozza, Giuseppe Vita, Mario Sabatelli

**Affiliations:** 1 Fondazione Policlinico Universitario A. Gemelli IRCCS, UOC Neurologia, 00168 Roma, Italy; marinaromozzi@gmail.com; 2 Dipartimento di Neuroscienze, Università Cattolica del Sacro Cuore, 00168 Roma, Italy; andrea.dp1988@gmail.com (A.D.P.); angela.romano12@gmail.com (A.R.); mario.sabatelli@unicatt.it (M.S.); 3 Centro Clinico NEMO-Fondazione Policlinico Universitario A. Gemelli IRCCS, 00168 Roma, Italy; giulia.bisogni@centrocliniconemo.it; 4 Dipartimento di Neuroscienze, Biomedicina e Scienze del Movimento, Università di Verona, 37134 Verona, Italy; davide.cardel@gmail.com (D.C.); tiziana.cavallaro@ospedaleuniverona.it (T.C.); gianmaria.fabrizi@univr.it (G.M.F.); matteo.tagliapietra@hotmail.com (M.T.); 5 Rare Neurodegenerative and Neurometabolic Diseases Unit, Department of Clinical Neurosciences, Fondazione IRCCS Istituto Neurologico Carlo Besta, 20133 Milano, Italy; silvia.fenu@istituto-besta.it (S.F.); Davide.Pareyson@istituto-besta.it (D.P.); 6 Department of Clinical and Experimental Medicine, University of Messina, 98125 Messina, Italy; lucagentile84@yahoo.it (L.G.); annamazzeo@yahoo.it (A.M.); russom@unime.it (M.R.); giuseppe.vita@unime.it (G.V.); 7 Dipartimento di Neuroscienze, Riabilitazione, Oftalmologia, Genetica e Scienze Materno Infantili (DINOGMI), Università degli studi di Genova, 16132 Genova, Italy; mgrandis@neurologia.unige.it (M.G.); saramassucco@libero.it (S.M.); aschenone@neurologia.unige.it (A.S.); 8 IRCCS San Martino, 16132 Genova, Italy; 9 Neuropathology Unit, Fondazione IRCCS Istituto Neurologico Carlo Besta, 20133 Milano, Italy; gianluca.marucci@istituto-besta.it; 10 Dipartimento di Neuroscienze, Scienze della Riproduzione e Oftalmologiche, Università Federico II di Napoli, 80131 Napoli, Italy; ste.tozza@gmail.com

**Keywords:** hATTR, nerve biopsy, amyloid, Congo red, axonal loss, polyneuropathy

## Abstract

Pathological evidence of amyloid on nerve biopsy has been the gold standard for diagnosis in hereditary transthyretin amyloidosis polyneuropathy (hATTR-PN) for a long time. In this article, we reviewed the pathological findings of a large series of sural nerve biopsies from a cohort of hATTR-PN patients, collected by different Italian referral centers. **Patients and Methods:** We reviewed clinical and pathological data from hATTR-PN patients, diagnosed and followed in five Italian referral centers for peripheral neuropathies. Diagnosis was formulated after a positive genetic test for transthyretin (*TTR*) mutations. Sural nerve biopsy was performed according to standard protocols. **Results:** Sixty-nine sural nerve biopsies from hATTR-PN patients were examined. Congo red positive deposits were found in 73% of cases. Only the Phe64Leu mutation failed to show amyloid deposits in a high percentage of biopsies (54%), as already described. Unusual pathological findings, such as myelin abnormalities or inflammatory infiltrates, were detected in occasional cases. **Conclusions:** Even if no longer indicated to confirm hATTR-PN clinical suspicion, nerve biopsy remains, in expert hands, a rapid and inexpensive tool to detect amyloid deposition. In Italy, clinicians should be aware that a negative biopsy does not exclude hATTR-PN, particularly for Phe64Leu, one of the most frequent mutations in this country.

## 1. Introduction

Hereditary transthyretin amyloidosis (hATTR) is an inherited, adult-onset, progressive disorder caused by mutations in the transthyretin (*TTR*) gene. The disease is characterized by extracellular deposition of amyloid insoluble fibrils in different organs, leading to a multisystem condition with prevalent peripheral nervous system and cardiac involvement [1].

The most typical presentations of hATTR are either a progressive, length-dependent, mixed sensory and motor peripheral polyneuropathy (hereditary transthyretin amyloidosis polyneuropathy, or hATTR-PN) associated with variable autonomic disturbances, formerly known as familial amyloid polyneuropathy (FAP), or an infiltrative cardiomyopathy (hereditary transthyretin amyloidosis cardiomyopathy, or hATTR-CM) [2]. The large majority of patients exhibits signs and symptoms of both nerve and heart involvement. hATTR is a life-threatening condition with a broad clinical presentation. The clinical course is marked by a progressive worsening, with a mean survival of 10 years since the onset of symptoms [1].

More than 120 *TTR* gene mutations have been identified as a cause of hATTR. Val30Met is the most frequent pathogenic variant and is primarily associated with neuropathy [3]. Phe64Leu is the most common mutation in southern Italy [4,5,6].

Depending on the geographic origin, a wide variation in age at onset and clinical presentation of the disorder is described. Generally, patients from endemic areas, such as Portugal, have an early-onset disease with initial involvement of small nerve fibers, while in non-endemic areas, many patients present with a late-onset progressive axonal polyneuropathy [7].

The diagnosis can be confirmed by demonstration of amyloid deposits in biopsy specimens, but identification of an amyloidogenic *TTR* variant by gene sequencing is the diagnostic gold standard [8]. Amyloid deposition can be found in different organs and tissues, such as skin, nerves, myocardium, abdominal fat, kidneys, labial salivary glands or the gastrointestinal tract [8,9]. Staining biopsy samples with Congo red, with characteristic green birefringence under polarized light, can reveal amyloid deposition. Amyloid typing by immunohistochemistry or mass spectrometry identifies the type of precursor protein [8,10].

Traditionally, biopsies of the affected tissues or organs were required for diagnosis. However, because of the patchy distribution of amyloid fibrils in tissues, a negative biopsy does not rule out the diagnosis, and multiple biopsies might be necessary in some cases. Furthermore, biopsy sensitivity depends on multiple factors, such as pathologist experience, the biopsied tissue, and the patient’s age [4,11,12].

The sensitivity of abdominal subcutaneous fat aspiration is approximately 50%, while a biopsy of salivary glands has a reported sensitivity in expert hands of 75–90% [13,14]. Conversely, nerve biopsy is usually considered a second-line investigation, and it can reach a sensitivity cited as high as 80% [15,16,17,18]. However, negative biopsies in hATTR remain a frequent cause of diagnostic delay [4,15].

In this article, we discuss the pathological findings of a large series of nerve biopsies of hATTR patients from Italian referral centers, in order to characterize the pathological nerve features of hATTR. 

## 2. Materials and Methods

### 2.1. Patients

Patients were retrospectively selected from those referred to five Italian centers representing referral centers for peripheral neuropathies and sharing an expertise in nerve biopsy (Fondazione Policlinico Universitario A. Gemelli IRCCS in Rome; Azienda Ospedaliera Universitaria of Verona; Azienda Ospedaliera Policlinico Universitario “G. Martino” of Messina; Fondazione IRCCS Istituto Neurologico Carlo Besta in Milan; Policlinico Universitario San Martino in Genoa). All patients with genetically confirmed hATTR that underwent nerve biopsy were included. In all patients, nerve biopsy preceded the genetic analysis, confirming a pathogenic mutation in *TTR* gene.

Clinical characteristics at the time of biopsy, including the polyneuropathy disability (PND) score [19], the presence of cardiomyopathy (defined as if a septum thickness >13 mm was present), autonomic involvement (gastrointestinal disturbance, postural hypotension, sexual dysfunction, heart rhythm disturbances), weight loss and other possible secondary causes of polyneuropathy, were collected by a questionnaire sent to all centers, and then reported.

Neuropathy was classified according to nerve conduction studies (NCS), performed before pathological examination, under the classification of axonal, demyelinating or mixed.

Results of fat needle aspiration or of other organ biopsies were reported if available.

### 2.2. Nerve Biopsy

Sural nerve biopsy was performed after obtaining informed consent, as previously described. Light and electron microscopy preparations, as well as teased fiber analysis, were performed according to standard methods [20]. Immunohistochemistry was performed in selected cases (see below). Sections were deparaffinized in xylene, rehydrated through decreasing concentrations of ethyl alcohol and then rinsed in distilled water. Antigen retrieval was performed in a 90 °C solution of 0.001 M EDTA buffer (pH 8.0) for 20 min. Endogenous peroxidase activity was quenched with 3% hydrogen peroxide in distilled water. Slides were incubated with antibodies recognizing CD3 (clone F7.2.38, 1:50, Dako), CD20 (clone L26, 1:50, Dako) and CD68 (clone KP1, 1:1000, Dako) for 1 h at room temperature. Bound antibodies were detected using Envision FLEX/HRP and 3–30-diaminobenzidine as chromogen (Dako). Slides were counterstained with haematoxylin (Sigma-Aldrich), dehydrated and mounted.

### 2.3. Statistical Analysis 

Statistical analysis of the data was performed by SPSS (Statistical Package for Social Science) version 24.0. Fisher’s 2-tailed exact test was used to compare numerical and nominal dichotomous variables, respectively. Significance was set at 0.05.

### 2.4. Ethics

Nerve biopsies are diagnostic tools in the setting of neuropathies, and all patients signed a written informed consent before the procedure. The study conforms to the ethical guidelines of the 1975 Declaration of Helsinki (6th revision, 2008), as reflected in a priori approval by the institution‘s human research committee.

## 3. Results

### 3.1. Clinical Results

A total of 67 patients (54 males and 13 females) underwent a sural nerve biopsy. The male-to-female ratio was 4.2. The mean age at disease onset was 64.1 years (median 67.0, standard deviation 10.8, range 30–81). Seven patients showed an early onset of the disease (<50 years). Mean disease duration at the time of biopsy was 2.9 years (median 2.0, standard deviation 3.1).

The geographical distribution of patients covered the whole Italy; 22 patients were from Sicily, 12 patients were from Lazio, 6 patients were from Apulia, 5 patients were from Calabria, 4 patients were from Campania and Lombardy, 3 patients were from Piedmont and Abruzzo, 2 patients were from Veneto and 1 patient was from Alto-Adige, Liguria, Emilia-Romagna and Tuscany. Two patients came from abroad (Macedonia and Holland, respectively).

The following *TTR* gene mutations were identified: Phe64Leu (26 patients); Val30Met (24 patients); Glu89Gln (6 patients); Tyr78Phe (3 patients); Glu62Lys (1 patient); Ala36Pro (1 patient); Ala34Thr (1 patient); Ile107Phe (1 patient); Pro33Val (1 patient); Ser50Arg (1 patient); Thr49Ala (1 patient); Val32Ala (1 patient).

Clinical disabilities, assessed by PND score, were as follows: 1 patient was in stage 1; 35 patients were in stage 2; 31 patients were in stage 3 (22 in stage 3A and 9 in stage 3B).

Considering NCS, an axonal neuropathy was present in 54 patients, a mixed neuropathy in 11 patients and a demyelinating neuropathy in 2 patients. NCS were performed 1–6 months before the sural nerve biopsy.

Cerebrospinal fluid examination was available for 28 patients (mean protein value 54.1 +/− 29.8 mg/mL, range 23–180). Seventeen cases showed a protein value > 50 mg/mL (mean value 68.7 +/− 30.1, range 52–180).

Cardiomyopathy was present in 47 out of 67 patients (70.1%), autonomic disturbances in 43 out of 67 (64.2%) and weight loss in 34 out of 67 (50.7%).

Considering the two most common mutations in our cohort, hypertrophic cardiomyopathy was more frequent in Val30Met when compared with Phe64Leu (20/24 vs 14/26, *p* = 0.0255), autonomic dysfunction was more frequent in Phe64Leu when compared with Val30Met (21/26 vs 10/24, *p* = 0.0080), and weight loss did not show frequency differences between Val30Met and Phe64Leu (10/24 vs 17/26).

Other possible secondary causes of polyneuropathy were identified in 13 cases: 4 patients (3 Val30Met and 1 Phe64Leu) with a concomitant monoclonal component of undetermined significance (MGUS), 3 patients (2 Phe64Leu and 1 Val30Met) with diabetes (one of them with a concomitant HCV infection), 3 patients (Thyr78Phe, Arg34Tyr, Phe64Leu) with cancer (lung, testis and lymphoma, respectively) not undergoing chemotherapy, 1 patient (Val32Ala) with renal failure, 1 patient (Val30Met) with alcohol abuse and 1 patient (Phe64leu) with a HBV infection.

### 3.2. Pathological Results

A total of 69 sural nerve biopsies were analyzed. Two male patients (Phe64Leu and Glu62Lys) underwent a second nerve biopsy, two years after the first one in both cases. The mean age at biopsy was 67.0 years (median 69.0, standard deviation 10.0, range 40–83).

**Figure 1:**
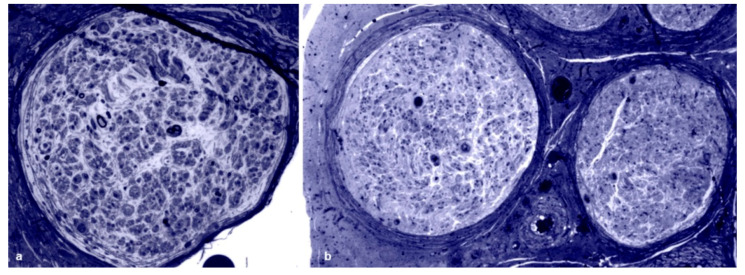
Sural nerve biopsies from hATTR patients. Semithin sections are stained with toluidine blue. (**a**) A biopsy from a 63-year-old Val30Met patient showing a marked loss of myelinated fibers. (**b**) A biopsy from a 74-year-old Phe64Leu patient with complete loss of myelinated fibers.

**Figure 2:**
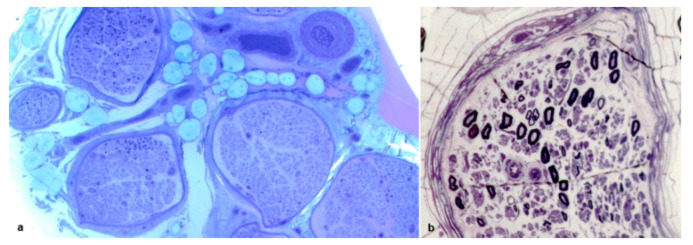
Sural nerve biopsies from a 71-year-old Tyr78Phe (**a**) and from a 68-year-old Phe64Leu (**b**) hATTR patients. Semithin sections stained with toluidine blue. An inhomogeneous interfascicular fiber loss is evident (**a**): in two fascicles only isolated myelin fibers were present while other fascicles show an asymmetrical fiber loss. An inhomogeneous intrafascicular fiber loss is present (**b**).

**Figure 3:**
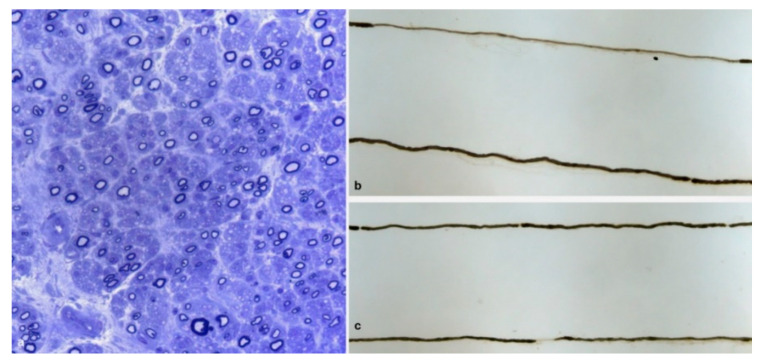
Sural nerve biopsy from a 69-year-old Tyr78Phe patient (**a**): semithin section stained with toluidine blue. A moderate fiber loss is observed. Many fibers are surrounded by thin myelin sheath. Several regenerating clusters are present. Teased fibers examination from a 44-year-old patient with Glu89Gln (**b**) and from a 73-year-old patient with Phe64Leu (**c**). A segmental demyelination is present in *b* while a paranodal demyelination is noted in *c*.

All biopsies showed evidence of axonal loss, which was severe in 42 cases, moderate in 15 and slight in 4 (Figure 1a,b). In 8 biopsies, we observed a complete loss of myelinated fibers (Figure 1b). Interestingly, in one biopsy, fiber loss was not homogeneously distributed among fascicles (Figure 2a). In one further case, an intrafascicular inhomogeneous fiber loss was detected (Figure 2b). Active axonal degeneration was found in 49 biopsies, with regenerating clusters in 22 (Figure 3a). Out of 22 biopsies, 5 showed regenerative clusters as possible evidence of a potential secondary cause of peripheral neuropathy.

Fibers surrounded by thin myelin sheaths were detected on semithin sections in 9 biopsies (Figure 3a) and were confirmed on teased fiber examination, which was available only for 36 biopsies, in 3 cases (Figure 3b,c). One further case showed only segmental demyelination on teased fiber analysis.

**Figure 4:**
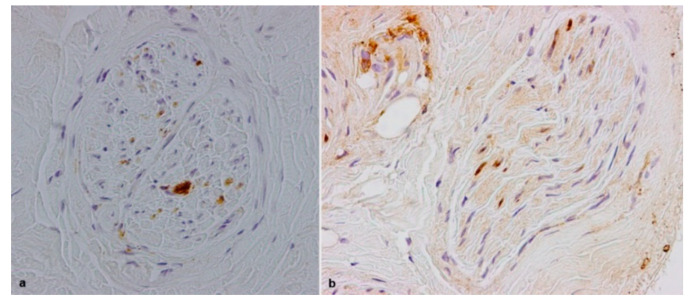
Sural nerve biopsies from a 61-year-old Val32Ala (**a**) and from a 74-year-old Val30Met (**b**) hATTR patients. Macrophages are depicted by CD68 (**a**). Some T lymphocytes are labelled by CD3 (**b**).

Inflammatory infiltrates, composed of histiocytes and some T lymphocytes, were observed in four biopsies (Figure 4a,b). In one of these biopsies (the patient with the Val32Ala mutation), a further possible secondary cause of polyneuropathy, namely renal failure, was also present.

**Figure 5:**
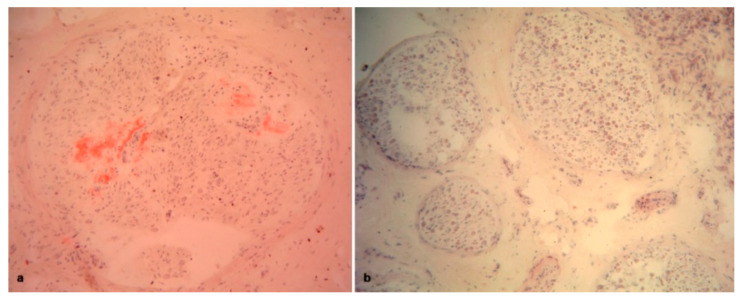
Sural nerve biopsies from a 76-year-old Val30Met (**a**) and from a 74-year-old Phe64Leu (**b**) hATTR patients. Congo red staining. Abundant amyloid congophilic deposits are evident in *a*, while no deposits are present in (**b**).

Congo red staining was positive in 50 of 69 cases (72.5%) (Figure 5a,b). Comparing the two most common mutations in our cohort, Congo red positive deposits were found in 21 of 24 Val30Met cases versus 13 of 27 Phe64Leu cases (*p* = 0.0035). We did not find any significant association between Congo red positive deposits and the clinical severity of polyneuropathy assessed by PND score, neither in the entire cohort (PND 1 and 2: 26 Congo red positive and 11 Congo red negative vs. PND 3A and 3B: 24 Congo red positive and 8 Congo red negative), nor considering the two most frequent mutations (Val30Met PND 2: 9 Congo red positive and 3 Congo red negative vs. Val30Met PND 3A and 3B: 12 Congo red positive and 0 Congo red negative; Phe64Leu PND 2: 6 Congo red positive and 6 Congo red negative vs. Phe64Leu PND 3A and 3B: 7 Congo red positive and 8 Congo red negative).

**Figure 6:**
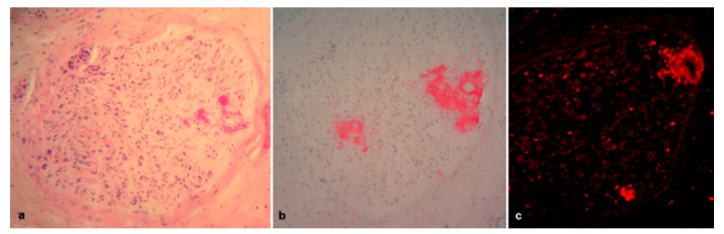
Sural nerve biopsy from a 73-year-old Val30Met hATTR patient. H&E staining (**a**) showed abundant TTR amyloid deposition confirmed also by Congo red staining (**b**) and by immunofluorescence with anti-TTR antibodies (**c**).

**Figure 7:**
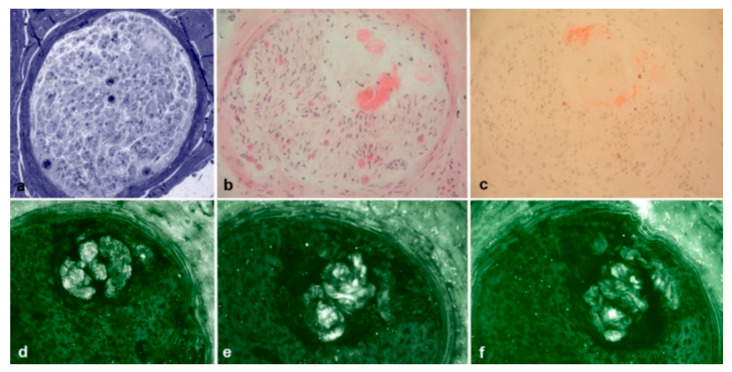
Sural nerve biopsy from a 76-year-old Val30Met hATTR patient. Semithin section stained with Toluidine blue (**a**) showed amyloid deposition confirmed also by H&E (**b**) and Congo red (**c**) staining. However, immunofluorescence with anti-TTR (**d**), anti-kappa light chain (**e**), and anti-lambda light chain (**f**) resulted not specific for TTR amyloidosis. No MGUS was detected in this patient.

Immunohistochemistry with anti-TTR, anti-kappa light chains and anti-lambda light chains was performed in 35 out of 50 Congo red positive biopsies, and a binding with anti-TTR antibodies was observed in all cases (Figure 6a–c). Interestingly, four cases also showed a cross-reaction with anti-light chains, but only in two of them a MGUS was detected (Figure 7a–f).

**Figure 8:**
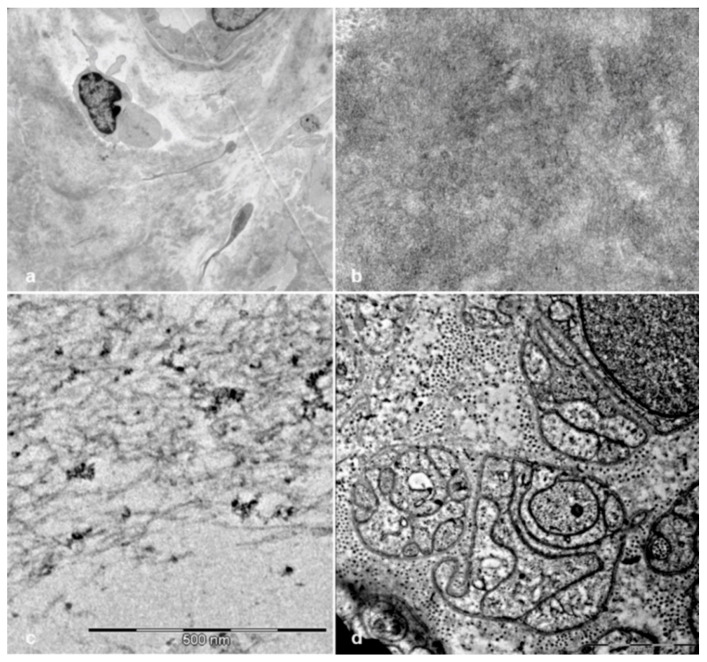
Electron microscope examination of sural nerve biopsies from hATTR patients. Ultrathin sections stained with uranyl acetate and lead citrate. Amyloid deposits are evident in a biopsy from a 65-year-old Val30Met patient (**a**,**b**). Amyloid from a 73-year-old Val30Met patient shows typical fibrillar structure (**c**). Unmyelinated fibers from a 74-year-old Phe64Leu patient are reduced and replaced by collagen pockets (**d**).

Electron microscope examination was available for 34 biopsies. In all of these cases, unmyelinated fibers were also reduced, and collagen pockets were observed (Figure 8d). Amyloid fibrils were detected in 11 cases (Figure 8a–c); 10 of these cases also showed amyloid deposits at Congo red staining while, in the remaining one with a Phe64Leu mutation, Congo red positive deposits were not found.

Abdominal fat needle aspiration was available for 20 out of 69 (29%) patients, and it yielded positive results for Congo red staining in 8 of them (8/20, 40%). Two patients with infiltrative cardiomyopathy underwent a myocardial biopsy, which proved positive for Congo red staining.

Detailed demographic, clinical and pathological findings of the entire hATTR cohort and of the most frequently detected mutations are summarized in Table 1.

## 4. Discussion

Diagnosis of hATTR is challenging, and frequently misdiagnosis results in delayed therapy [4,21]. For many years, pathological evidence of amyloid deposition had been the gold standard for diagnosis, but in the last few years, also considering the introduction of new possible therapeutic approaches, genetic testing has become the first option after a clinical suspicion.

However, nerve biopsy remains a privileged instrument for a possible diagnosis in atypical cases in which a different clinical diagnosis was suspected, or to speculate about the mechanism of nerve damage induced by amyloid fibrils [8].

We examined a wide sample of nerve biopsies from hATTR patients coming from different Italian referral centers. The demographic characteristics of our cohort reflect those of classical late-onset hATTR, with a mean age of onset over 60 years and a male prevalence. We observed few early-onset cases, namely those carrying the Glu89Gln variant, the most frequent mutation in Sicily [17].

Regarding observed mutations, we confirmed that Val30Met was not the most common variant in Italy, with Phe64Leu being the most frequently found one in this cohort [5,6]. Although our data were focused on hATTR nerve biopsies, results were similar to those of the ATTRv amyloidosis Italian Registry [6], with the absence of Ile68Leu that classically shows a cardiological phenotype without nerve involvement.

The clinical spectrum included all the possible manifestations of hATTR. Hypertrophic cardiomyopathy was common in Val30Met, while autonomic dysfunction was frequent in Phe64Leu.

Regarding the polyneuropathy observed in this cohort, an axonal neuropathy was obviously the most common feature; it was found in 80.6% of patients and perfectly reflected the axonal loss showed by nerve biopsies, which ranged from moderate to complete loss in 94.2% of biopsies. In 16.4% of patients, a mixed polyneuropathy was described. In these cases, demyelinating findings were probably secondary to axonal loss, rather than caused by direct damage to the myelin, as frequently observed in other neuropathies [22,23]. However, since TTR is expressed in Schwann cells, a direct toxic effect on these cells, and on the nearby dorsal root ganglia, cannot be excluded [24,25]. There is progressive evidence that the oligomers of amyloidogenic proteins play a key role in mediating toxicity in other common neurodegenerative diseases, including Alzheimer’s disease and Parkinson’s disease [26]. Hence, biochemical stresses may be responsible for Schwann cell damage in patients with hATTR, in addition to the mechanical stress resulting from the formation of amyloid fibrils.

Interestingly, in two patients, a demyelinating neuropathy, according to EFNS/PNS criteria [27], was reported. In such cases, carrying the Val30Met and Thy78Phe mutations, respectively, a mechanic demyelination due to compression by amyloid deposition on nerve fibers [11,28] or a superimposed inflammatory process, as described in other cases of inherited neuropathies, could be hypothesized [25,29,30,31]. Supporting both hypotheses, the nerve biopsy of the patient with Tyr78Phe showed a moderate axonal loss with inhomogeneous distribution among fascicles and Congo red positive amyloid deposits, while the cerebrospinal fluid revealed a moderate protein increase (56 mg/dL). Conversely, the nerve biopsy of the patient with Val30Met showed a moderate axonal loss with many fibers surrounded by thin myelin sheaths and Congo red positive amyloid deposits, while the cerebrospinal fluid revealed a normal protein level (30 mg/dL).

Myelin abnormalities were observed in ten biopsies. Unfortunately, cerebrospinal fluid examination was available only for the patients previously described. However, in one of these biopsies, inflammatory infiltrates were also found, and we observed inflammatory infiltrates in a further three biopsies, supporting the hypothesis of a superimposed inflammation [29,30]. Furthermore, an increase of protein in the cerebrospinal fluid may be found in hATTR [21], and it was present in about 60% of the exams available for our cohort. However, we cannot exclude that a protein increase in the cerebrospinal fluid examination is simply a consequence of radicular damage more than the sign of a superimposed inflammatory process. Uncommon pathological findings with myelin abnormalities and the presence of hyperproteinorrachia may be responsible for the frequent misdiagnosis of inflammatory neuropathies [21].

Regarding the sensitivity of nerve biopsies to detect amyloid, Congo red positive deposits were found in 72.5% of our biopsies. This percentage was higher if compared with abdominal fat needle aspiration, which was positive only in 40% of cases in our cohort. However, we should consider that this test was performed in 20 patients only. Regarding the different mutations, previous studies demonstrated that Phe64Leu mutation is not always associated with pathological deposits of amyloid in nerve biopsies or abdominal fat needle aspiration [4,15]. Analysis of our cohort confirms that Congo red positive deposits are less common with Phe64Leu mutations than with Val30Met mutations. The Congo red positivity of 87% with the Val30Met mutation, on the other hand, was similar to that reported in a recently published paper [32]. In contrast to this study, we did not find any significant correlation between the presence of Congo red deposits and the clinical severity of polyneuropathy, as assessed by PND score [32]. The proportion of early-onset patients in our cohort might explain this difference.

Immunohistochemistry with anti-TTR antibodies was able to confirm the nature of the deposits in all cases with Congo red positive deposits. However, we observed a cross-reaction with anti-lambda light chains in four biopsies. Two cases did not show MGUS at serum immunofixation. A possible cross-reaction among antibodies for amyloid typing has been described [11], and it confirms the importance of excluding hATTR through genetic testing when amyloid light-chain (AL) amyloidosis is suspected. On the other hand, in the other two cases, a MGUS was present, and a concomitant AL was diagnosed, confirming the importance of typing amyloid deposits [12].

Considering unmyelinated fibers, electron microscope examination, performed in half of the biopsies, also confirmed their involvement in late-onset hATTR, as demonstrated by other techniques [33,34,35].

## 5. Conclusions

Even if no longer indicated to confirm hATTR-PN clinical suspicion, nerve biopsy remains, in expert hands, a rapid and inexpensive tool to detect amyloid deposition. However, in Italy, considering the prevalence of Phe64Leu, clinicians should be aware of the relevant rate of false negatives for this biopsy, as also happens for myocardial bone scintigraphy [36]. Unusual pathological findings, such as myelin abnormalities and inflammatory infiltrates, may be found, and they do not exclude this diagnosis.

## Figures and Tables

**Table 1:** Demographic, clinical and pathological findings of the entire hATTR cohort and of the most frequently detected mutations.

	All Patients	Phe64Leu	Val30Met	Glu89Gln	Tyr68Phe
**Number of patients**	67	26	24	6	3
**M/F**	54/13	22/4	20/4	5/1	3/0
**Age at onset**	64.1	68.7	67.3	48.2	63.3
**Age at biopsy**	67.0	71.1	69.6	55.3	64.7
**Disease duration at biopsy (years)**	2.9	2.4	2.3	7.1	3.4
**Origin**					
**Northern Italy**	13/67 (19.4%)	1/26 (3.8%)	8/24 (33.3%)	0/6 (0.0%)	2/3 (66.7%)
**Central Italy**	19/67 (28.3%)	2/26 (7.6%)	15/24 (62.5%)	0/6 (0.0%)	0/3 (0.0%)
**Southern Italy**	33/67 (49.3%)	23/26 (88.6%)	0/24 (0.0%)	6/6 (100%)	1/3 (33.3%)
**Other countries**	2/67 (3.0%)	0/26 (0.0%)	1/24 (4.2%)	0/6 (0.0%)	0/3 (0.0%)
					
**Polyneuropathy**					
**Axonal**	54/67 (80.6%)	23/26 (88.5%)	21/24 (87.5%)	4/6 (66.7%)	1/3 (33.3%)
**Mixed**	11/67 (16.4%)	3/26 (11.5%)	2/24 (8.3%)	2/6 (33.3%)	1/3 (33.3%)
**Demyelinating**	2/67 (3.0%)	0/26 (0.0%)	1/24 (4.2%)	0/6 (0.0%)	1/3 (33.3%)
					
**Cardiomyopathy**	47/67 (70.2%)	14/26 (53.9%)	20/24 (83.4%)	5/6 (83.3%)	1/3 (33.3%)
**Autonomic disturbances**	43/67 (64.2%)	21/26 (80.8%)	10/24 (41.7%)	5/6 (83.3%)	2/3 (66.7%)
**Weight loss**	34/67 (50.8%)	17/26 (65.4%)	10/24 (41.7%)	2/6 (33.3%)	1/3 (33.3%)
					
**Congo red positive abdominal fat needle aspiration**	8/20 (40.0%)	1/6 (16.6%)	6/11 (54.5%)	-	1/1 (100%)
					
**Number of biopsies**	69	27	24	6	3
**Congo red positive nerve biopsy**	50/69 (72.5%)	13/27 (48.2%)	21/24 (87.5%)	5/6 (83.3%)	2/3 (66.7%)
**Axonal loss**					
**Slight**	4/69 (5.8%)	0/27 (0.0%)	0/24 (0.0%)	0/6 (0.0%)	1/3 (33.3%)
**Moderate**	15/69 (21.7%)	1/27 (3.7%)	7/24 (29.2%)	2/6 (33.3%)	2/3 * (66.7%)
**Severe**	42/69 (60.9%)	24/27 (88.9%)	13/24 (54.2%)	4/6 (66.7%)	0/3 (0.0%)
**Total**	8/69 (11.6%)	2/27 (7.4%)	4/24 (16.7%)	0/6 (0.0%)	0/3 (0.0%)
					
**Myelin abnormalities**	10/69 (14.5%)	4/27 (14.8%)	2/24 (8.3%)	1/6 (16.7%)	2/3 (66.7%)
**Inflammatory infiltrates**	4/69 (5.8%)	1/27 (3.7%)	0/24 (0.0%)	0/6 (0.0%)	0/3 (0.0%)

***Legend to the Table:*** Northern Italy includes Lombardy, Piedmont, Veneto, Alto-Adige, Liguria, Emilia-Romagna and Tuscany; Central Italy includes Lazio, Campania and Abruzzo; Southern Italy includes Sicily, Apulia and Calabria; other countries include Macedonia and Holland. All Glu89Gln cases are from Sicily; all 11 Val30Met cases are from Lazio. * One of these two biopsies showed an inhomogeneous interfascicular fiber loss.
